# The relationship between work-family conflict and occupational stress in emergency department nurses: a cross-sectional correlational study

**DOI:** 10.3389/fpubh.2026.1892914

**Published:** 2026-07-27

**Authors:** Pei Wang, Hao Zhang, Xiaoli Chen, Le Tong

**Affiliations:** 1 Department of Emergency Medicine, West China Hospital, Sichuan University/West China School of Nursing, Sichuan University, Chengdu, China; 2 Institute of Disaster Medicine, Sichuan University, Chengdu, China; 3 Nursing Key Laboratory of Sichuan Province, Chengdu, China

**Keywords:** cross-sectional study, emergency department nurses, influencing factors, occupational stress, work-family conflict

## Abstract

**Background:**

Emergency department (ED) nurses work in high-stress environments and face multiple role pressures simultaneously, making occupational stress and work-family conflict increasingly prominent occupational health concerns in this population. However, the prevalence and correlates of these two constructs have not been systematically examined in the Chinese ED nursing context.

**Objectives:**

This study aimed to describe the current status of occupational stress and work-family conflict among emergency department nurses, and to examine the association between the two constructs and identify potential influencing factors.

**Methods:**

A cross-sectional design was adopted. From December 26, 2023, to January 18, 2024, a total of 1,540 emergency department (ED) nurses from 30 tertiary hospitals across 20 provinces, autonomous regions, and municipalities in China were recruited using a stratified cluster sampling method. Occupational stress and work-family conflict were assessed using a self-designed general information questionnaire, the occupational stress questionnaire, and the Work-Family Behavioral Role Conflict Scale. Data were analyzed using SPSS 25.0, employing descriptive statistics, Pearson correlation analysis, and multiple linear regression to examine the associations between occupational stress and work-family conflict.

**Results:**

A total of 1,540 valid questionnaires were included in the final analysis. The majority of participants were female, aged 30–39 years, held a bachelor's degree, were married with children, and had a junior professional title. The mean total occupational stress score was 55.55 ± 16.78, with dimension scores of 19.22 ± 5.35 for effort, 23.72 ± 9.10 for reward, and 12.60 ± 4.74 for overcommitment. The mean total work-family conflict score was 42.48 ± 16.21, with subscale scores of 18.27 ± 6.88 for work-to-family conflict and 24.21 ± 10.75 for family-to-work conflict. Pearson correlation analysis revealed that the total work-family conflict score and both of its dimensions were positively correlated with the total occupational stress score and all three dimensions, with correlation coefficients ranging from 0.462 to 0.664 (all *P* < 0.001), indicating moderate-to-strong associations between work-family conflict and occupational stress. Multiple linear regression identified the following factors as independently associated with occupational stress: bachelor's degree or above (*B* = 1.928, 95% CI: 0.021–3.835, *P* = 0.048), senior professional title (*B* = 4.744, 95% CI: 1.046–8.441, *P* = 0.012), weekly working hours of 41–48 h (*B* = 1.585, 95% CI: 0.292–2.877, *P* = 0.016), 49–58 h (*B* = 5.419, 95% CI: 3.073–7.765, *P* < 0.001), and ≥59 h (*B* = 7.353, 95% CI: 4.144–10.562, *P* < 0.001), monthly night shift frequency >8 times/month (*B* = 2.975, 95% CI: 0.689–5.261, *P* = 0.011), work-to-family conflict (*B* = 1.244, 95% CI: 1.123–1.365, *P* < 0.001), and family-to-work conflict (*B* = 0.319, 95% CI: 0.243–0.395, *P* < 0.001).

**Conclusions:**

Work-family conflict is significantly associated with elevated occupational stress among emergency department nurses. Nurses with higher education levels, senior professional titles, longer working hours, and higher monthly night shift frequency tend to report higher levels of occupational stress. These findings suggest that nursing managers may consider controlling working hours, monitoring night shift frequency (>8 shifts per month) and fostering a family-supportive organizational culture as potential strategies to alleviate occupational stress in this population. However, given the cross-sectional design of this study, these associations should not be interpreted as causal, and the proposed strategies should be validated through future longitudinal research.

## 1 Introduction

Occupational stress (OS), also referred to as job stress, is defined as the adverse physiological and psychological responses that occur when job demands do not match an individual's abilities, or when resources and needs remain unmet (1). Due to sustained and substantial occupational pressure, healthcare workers have become one of the populations with the highest prevalence of occupational stress. A recent review indicated that over 52.9% of healthcare workers in Ethiopia experience occupational stress (2). Kakemam et al. (3) reported that 78.4% of nurses experienced occupational stress; in another study conducted in Iran, the reported prevalence of occupational stress among nurses was 49.5% (4). A systematic review further estimated that the economic burden of occupational stress ranges from US$221.13 million to US$187 billion, with 70%−90% of productivity-related losses attributed to this condition (5).

The work environment of the emergency department (ED) is characterized by multiple stressors, including high unpredictability, frequent emergencies, sustained heavy workloads, and pronounced tension in nurse-patient relationships (6, 7). ED nurses are not only required to cope with high-intensity tasks and unpredictable fluctuations in patient volume over the long term, but are also frequently exposed to traumatic resuscitation events, the risk of workplace violence, circadian rhythm disruption due to shift work, and extreme situations involving life-and-death decision-making (8–10). Such sustained high-effort conditions place ED nurses at particularly high risk for occupational stress. A systematic review has demonstrated that the prevalence of occupational stress among ED nurses is notably high, with over 60% of nurses affected (11). A cross-sectional study from China has reported a similarly concerning finding, with a detection rate of 59.66% (12). Collectively, these findings underscore that occupational stress among ED nurses demands urgent attention and intervention.

Work-family conflict arises when individuals are unable to fulfill family responsibilities due to work demands, or when family burdens interfere with work performance (13). The very nature of clinical nursing imposes a structural encroachment on family role fulfillment, such that nurses face persistent challenges in carrying out family obligations, participating in child rearing, and maintaining intimate relationships. Pien et al. (14) demonstrated that work-family conflict perceived by nurses is not only prevalent but also chronically sustained at high levels. Zandian et al. (15) confirmed that up to 93% of nurses reported moderate-to-severe work-family conflict. A large-scale survey of 70,932 nurses in China conducted by Chen et al. (16) further confirmed that work-family conflict has become a critical reality for this population.

According to role stress theory, role conflict arises when individuals face incompatible expectations from multiple roles they occupy simultaneously. Work-family conflict, as a chronic role stressor, makes it difficult for clinical nurses to attend to family obligations due to frequent shift work and overtime (17). Concurrently, the guilt and frustration arising from impaired family role fulfillment may add to the psychological burden already experienced in the work domain (18). This perspective provides a theoretical foundation for understanding the association between work-family conflict and occupational stress.

Nevertheless, existing studies on ED nurses have predominantly focused on endogenous workplace stressors, while the association between work-family conflict and occupational stress remains insufficiently explored. Given this gap, the present study incorporates work-family conflict into the analytical framework, aiming to describe the current status of occupational stress and work-family conflict among ED nurses, to examine the association between the two constructs, and to identify key demographic and occupational factors associated with occupational stress. The findings are expected to provide an empirical basis for developing intervention strategies that integrate both occupational support and family-friendly policies.

## 2 Methods

### 2.1 Study design

A multicenter cross-sectional study design was adopted.

### 2.2 Participants

This study employed a stratified cluster sampling method and was conducted nationwide from December 26, 2023, to January 18, 2024. Stratification was based on China's standard geographical division criteria, dividing the country into seven major regions: Northeast, North China, East China, Central China, South China, Southwest, and Northwest. Within each region, 2–3 tertiary hospitals were selected from provincial capitals, prefecture-level cities, and municipalities directly under the central government as cluster sampling units. Ultimately, 30 tertiary hospitals across 20 provinces, autonomous regions, and municipalities were included. All eligible emergency department nurses in each participating hospital were invited to complete the survey. A total of 1,551 questionnaires were returned. Of these, 11 were excluded due to incomplete responses or patterned answering, yielding 1,540 valid questionnaires and an effective response rate of 99.29%.

#### 2.2.1 Inclusion criteria

(1) Holding a registered nursing license and currently employed; (2) Having worked continuously in the ED for at least one year; (3) Providing informed consent and voluntarily participating in the study.

#### 2.2.2 Exclusion criteria

Nurses still undergoing standardized training programs, those on advanced studies, or those temporarily absent from work due to sick leave, maternity leave, or other reasons.

An *a priori* sample size calculation was performed using G^*^Power 3.1 for multiple linear regression analysis examining the association between work-family conflict and occupational stress among emergency nurses. With an anticipated medium effect size (*f*^2^ = 0.10), 15 predictor variables, a significance level of α = 0.05, and a statistical power of 0.90, the estimated minimum required sample size was 204 participants. To account for the design effect (DEFF = 1.5) due to the cluster sampling approach and a anticipated invalid response rate of 10%, the final target sample size was adjusted to 340 participants.

### 2.3 Instruments

#### 2.3.1 General information questionnaire

A self-designed general information questionnaire was used to collect participants' demographic and occupational characteristics, including age, sex, education level, marital status, parental status, professional title, years of work experience, monthly income, weekly working hours, and frequency of night shifts.

#### 2.3.2 Occupational stress scale

Occupational stress was measured using the effort-reward imbalance (ERI) scale developed by Siegrist et al. (19) and translated into Chinese by Dai et al. (20). In this study, the scale served solely as an instrument for assessing the level of occupational stress and was not intended for testing the theoretical mechanism of effort-reward imbalance. The scale consists of 22 items across three dimensions: effort (6 items), reward (11 items), and overcommitment (5 items). Each item is rated on a 5-point Likert scale (1–5), and the score for each dimension is calculated as the sum of its constituent items. In this study, the Cronbach's α coefficient for the overall scale was 0.957, with values of 0.894, 0.952, and 0.922 for the three dimensions, respectively.

#### 2.3.3 Work–family conflict scale

Work–family conflict among emergency department nurses was measured using the Work–Family Behavioral Role Conflict Scale (WFBRC-S), originally developed by Clark et al. (21) and subsequently translated and revised by Zhang (22). The WFBRC-S consists of 19 items divided into two dimensions: work-to-family behavioral role conflict and family-to-work behavioral role conflict. Each item is rated on a 5-point Likert scale, with all items positively scored; higher scores indicate more severe role conflict. The work-family conflict scale demonstrated excellent internal consistency, with a Cronbach's α of 0.969 for the total scale and 0.941 and 0.975 for the two subscales, respectively.

### 2.4 Data collection and quality control

An electronic questionnaire was designed and generated using the Wenjuanxing platform. The first page of the questionnaire included an informed consent form, followed by the survey items. After obtaining informed consent, the questionnaire link and QR code were distributed via WeChat groups to ED nurses who met the inclusion criteria, accompanied by an explanation of the study purpose. Participation was voluntary, and responses were collected anonymously. To prevent duplicate submissions, the same IP address was restricted to a single response. Upon retrieval of the questionnaires, logical checks were performed individually. Invalid questionnaires—those with identical responses to all items or contradictory information (e.g., obvious discrepancies between age and years of experience or professional title)—were excluded.

### 2.5 Statistical analysis

Data were analyzed using SPSS 26.0. Normally distributed continuous variables were presented as mean ± standard deviation (x ± s), and comparisons between two groups were conducted using the independent samples t-test, while comparisons among multiple groups were performed using one-way analysis of variance (ANOVA). Categorical variables were described using frequencies and percentages. Pearson correlation analysis was employed to examine the associations between work-family conflict and each dimension of occupational stress. Multiple linear regression analysis was conducted with the total occupational stress score as the dependent variable. General demographic variables that were statistically significant in univariate analyses, along with the dimensions of work-family conflict, were entered as independent variables to identify independent factors associated with occupational stress. Variables entered into the regression model were required to meet a variance inflation factor (VIF) < 10. A two-tailed *P*-value < 0.05 was considered statistically significant.

### 2.6 Ethical considerations

This study was conducted in accordance with the Declaration of Helsinki. Ethical approval was obtained from the Ethics Committee of West China Hospital of Sichuan University (Approval No. 2024.309). All participants provided written informed consent prior to participation. Anonymity and confidentiality of responses were guaranteed throughout the study. Data were stored securely and accessed only by the research team.

## 3 Results

### 3.1 Characteristics of study participants

A total of 1,540 emergency department nurses were included in this study. Their demographic and work-related characteristics are presented in Table 1. The majority of participants were aged 30–39 years (47.7%). Female nurses accounted for 78.6% and male nurses for 21.4%. Of the sample, 63.6% were married and 53.9% had children. In terms of education, 87.9% held a bachelor's degree or above. Regarding professional titles, the majority held junior titles (58.9%). Nurses with ≤ 10 years of work experience comprised 63.9% of the sample. Weekly working hours of 41–48 h were most common (50.6%). Among the participants, 88.2% were required to work night shifts. Regarding monthly night shift frequency, 15.5% reported 1–4 shifts, 42.8% reported 5–8 shifts, and 29.9% reported >8 shifts per month.

**Table 1:** Characteristics of the participants (*n* = 1,540).

Variables	Categories	*N*	%
Age	20–29 years	582	37.8
	30–39 years	734	47.7
	40–49 years	183	11.9
	≥50 years	41	2.7
Gender	Female	1,211	78.6
	Male	329	21.4
Marital status	Unmarried	560	36.4
	Married	980	63.6
Number of children	No	710	46.1
	Yes	830	53.9
Educational level	Associate degree	186	12.1
	Bachelor's degree or above	1,354	87.9
Professional title	Junior RN	907	58.9
	Middle RN	574	37.3
	Senior RN	59	3.8
Years of nursing experience	≤ 5years	491	31.9
	6–10 years	493	32.0
	11–15 years	300	19.5
	16–20 years	121	7.8
	>20 years	135	8.8
Working hours per week	≤ 40 h per week	577	37.5
	41 ~ 48 h per week	780	50.6
	49 ~ 58 h per week	123	8.0
	≥59 h per week	60	3.9
Night shift	No	181	11.8
	Yes	1,359	88.2
Number of night shift/month	0	181	11.8
	1–4 times per month	239	15.5
	5–8 times per month	659	42.8
	>8 times per month	461	29.9
Monthly income (yuan)	< 4,000 yuan	57	3.7
	4,000–5,999 yuan	149	9.7
	6,000–7,999 yuan	250	16.2
	8,000–9,999 yuan	368	23.9
	≥10,000 yuan	716	46.5
Smoking status	No	1,453	94.4
	Yes	87	5.6
Drinking status	No	1,355	88.0
	Yes	185	12.0

RN, registered nurse.

### 3.2 Descriptive analysis of occupational stress and work-family conflict

The total scores and dimension scores of both occupational stress and work-family conflict conformed to a normal distribution. The mean total occupational stress score was 55.55 ± 16.78, with dimension scores of 19.22 ± 5.35 for effort, 23.72 ± 9.10 for reward, and 12.60 ± 4.74 for overcommitment. The mean scores for the two dimensions of work-family conflict were 18.27 ± 6.88 for work-to-family behavioral role conflict and 24.21 ± 10.75 for family-to-work behavioral role conflict. Descriptive statistics for all study variables are summarized in Table 2.

**Table 2:** Analysis of occupational stress and work-family conflict scores.

Variables	Dimension	Min	Max	Mean	SD
Occupational stress	Effort	6	30	19.22	5.35
	Reward	11	55	23.72	9.10
	Overcommitment	5	25	12.60	4.74
	Total score	22	110	55.55	16.78
Work-to-family role conflict	Work-to-family conflict	7	35	18.27	6.88
	Family-to-work conflict	12	60	24.21	10.75
	Total scores	19	95	42.48	16.21

SD, standard deviation.

### 3.3 Pairwise correlation analysis of total scores and sub-dimensions between occupational stress and work-family conflict

Pearson correlation analysis was performed to examine the associations between work-family conflict and occupational stress. The results are presented in Table 3. The total work-family conflict score was significantly positively correlated with the total occupational stress score (*r* = 0.664, *P* < 0.01), indicating a moderate-to-strong association between the two constructs. At the dimension level, work-to-family conflict demonstrated significant positive correlations with the total occupational stress score (*r* = 0.679, *P* < 0.01), as well as with the effort, reward, and overcommitment dimensions (*r* = 0.571, 0.565, and 0.675, respectively; all *P* < 0.01). These coefficients, ranging from 0.565 to 0.679, indicate moderate-to-strong associations. Family-to-work conflict was also significantly positively correlated with the total occupational stress score (*r* = 0.567, *P* < 0.01), and with the effort, reward, and overcommitment dimensions (*r* = 0.331, 0.580, and 0.521, respectively; all *P* < 0.01). The correlation between family-to-work conflict and the effort dimension (*r* = 0.331) was relatively weaker yet remained statistically significant, whereas the correlations with the reward and overcommitment dimensions fell within the moderate range.

**Table 3:** Pairwise correlation analysis of total scores and sub-dimensions between occupational stress and work-family conflict.

Variables	Occupational stress				Work-to-family role conflict		
	Effort	Reward	Overcommitment	Total scores	Work-to-family conflict	Family-to-work conflict	Total scores
Effort	1	–	–	–	–	–	–
Reward	0.501^**^	1	–	–	–	–	–
Overcommitment	0.662^**^	0.759^**^	1	–	–	–	–
Total scores	0.777^**^	0.916^**^	0.905^**^	1	–	–	–
Work-to-family conflict	0.571^**^	0.565^**^	0.675^**^	0.679^**^	1	–	–
Family-to-work conflict	0.331^**^	0.580^**^	0.521^**^	0.567^**^	0.676^**^	1	–
Total scores	0.462^**^	0.624^**^	0.632^**^	0.664^**^	0.873^**^	0.950^**^	1

^**^P < 0.01.

## 4 Factors associated with occupational stress

### 4.1 Univariate analysis

Univariate analyses were conducted with occupational stress as the dependent variable and general demographic characteristics and the dimensions of work-family conflict as independent variables. The results showed that age, marital status, parental status, education level, professional title, years of work experience, weekly working hours, and frequency of night shifts were significantly associated with occupational stress (all *P* < 0.05), as detailed in Table 4.

**Table 4:** Results of univariate analysis of occupational stress.

Variables	Categories	x̄ ±s	*t*/*F*	*P*
Age	20–29 years	52.45 ± 17.40	11.674	< 0.001
	30–39 years	57.41 ± 16.04		
	40–49 years	58.31 ± 16.29		
	≥50 years	53.76 ± 16.29		
Gender	Female	55.64 ± 16.33	0.418	0.676
	Male	55.18 ± 18.35		
Marital status	Unmarried	53.12 ± 16.94	−4.307	< 0.001
	Married	56.93 ± 16.54		
Number of children	No	53.89 ± 16.93	−3.598	< 0.001
	Yes	56.96 ± 16.52		
Educational level	Associate degree	49.68 ± 17.88	−5.128	< 0.001
	Bachelor's degree or above	56.35 ± 16.47		
Professional title	Junior RN	53.51 ± 16.94	19.955	< 0.001
	Middle RN	57.92 ± 16.07		
	Senior RN	63.76 ± 15.74		
Years of nursing experience	≤ 5years	51.97 ± 17.43	8.877	< 0.001
	6–10 years	56.68 ± 16.39		
	11–15 years	57.31 ± 15.85		
	16–20 years	59.13 ± 16.36		
	>20 years	57.26 ± 16.21		
Working hours per week	≤ 40h per week	51.95 ± 15.52	24.032	< 0.001
	41 ~ 48h per week	56.37 ± 15.94		
	49 ~ 58h per week	62.48 ± 19.24		
	≥59h per week	65.15 ± 23.27		
Night shift	No	55.56 ± 16.87	0.107	0.915
	Yes	55.42 ± 16.12		
Number of night shift	0	55.42 ± 16.12	7.604	< 0.001
	1–4 times per month	52.53 ± 14.57		
	5–8 times per month	54.70 ± 16.96		
	>8 times per month	58.37 ± 17.47		
Monthly income (yuan)	< 4,000 yuan	58.61 ± 20.86	1.108	0.351
	4,000–5,999 yuan	56.17 ± 19.70		
	6,000–7,999 yuan	56.41 ± 17.84		
	8,000–9,999 yuan	55.77 ± 17.26		
	≥10,000 yuan	54.75 ± 15.03		
Smoking status	No	55.37 ± 16.66	−1.689	0.092
	Yes	58.49 ± 18.46		
Drinking status	No	55.35 ± 16.75	−1.234	0.217
	Yes	56.97 ± 17.00		

### 4.2 Multivariable analysis

To assess the potential clustering effect of data at the hospital level, we calculated the intraclass correlation coefficient (ICC) for the total occupational stress score using a one-way random-effects model. The estimated ICC was 0.087, indicating that only 8.7% of the total variance was attributable to between-hospital differences. Given this low ICC value, we considered the clustering effect negligible and proceeded with ordinary multiple linear regression. All regression models were adjusted for demographic and work-related variables identified in univariate analyses. Multiple linear regression analysis was conducted with the total occupational stress score as the dependent variable. Variables that were statistically significant in the univariate analyses, along with the dimensions of work-family conflict, were entered into the model. The regression model was statistically significant (*F* = 78.247, *P* < 0.001), with an adjusted *R*^2^ of 0.501, indicating that the model explained 50.1% of the variance in occupational stress among emergency department nurses. The Durbin–Watson statistic was 1.945, which is close to 2, suggesting satisfactory independence of residuals and an adequate model fit (see Supplementary Table 1).

Multivariable analysis revealed that holding a bachelor's degree or above, having a senior professional title, extended weekly working hours (41–48 h, 49–58 h, and ≥59 h), night shift frequency >8 times per month, work-to-family conflict, and family-to-work conflict were independent factors associated with occupational stress (all *P* < 0.05). Among these, work-to-family conflict (Beta = 0.510, *P* < 0.001) and family-to-work conflict (Beta = 0.204, *P* < 0.001) demonstrated the strongest positive associations with occupational stress. Occupational stress scores increased progressively with longer weekly working hours and higher night shift frequency (see Table 5 for details). Collinearity diagnostics showed that all tolerance values were > 0.1 and all variance inflation factors (VIF) were below 10 (ranging from 1.098 to 5.945), indicating no significant multicollinearity among the independent variables (see Supplementary Table 1).

**Table 5:** Results of multiple linear regression analysis.

Variables		Unstandardized coefficient		Standardized coefficient	t	*P*	95%CI	
		*B*	SE	*Beta*			Lower	Upper
Constant		19.453	1.571	–	12.379	< 0.001	16.371	22.536
Age	20–29 years	–	–	–	–	–	–	–
	30–39 years	1.444	1.296	0.043	1.115	0.265	−1.098	3.986
	40–49 years	−0.770	2.193	−0.015	−0.351	0.726	−5.071	3.531
	≥50 years	−2.599	3.130	−0.025	−0.830	0.407	−8.739	3.542
Marital status	Unmarried	–	–	–	–	–	–	–
	Married	1.290	1.090	0.037	1.184	0.237	−0.848	3.428
Number of children	No	–	–	–	–	–	–	–
	Yes	−2.112	1.088	−0.063	−1.940	0.053	−4.246	0.023
Educational level	Associate degree	–	–	–	–	–	–	–
	Bachelor's degree or above	1.928	0.972	0.037	1.983	0.048	0.021	3.835
Professional title	Junior RN	–	–	–	–	–	–	–
	Middle RN	0.500	0.816	0.014	0.613	0.540	−1.100	2.100
	Senior RN	4.744	1.885	0.054	2.517	0.012	1.046	8.441
Years of nursing experience	≤ 5years	–	–	–	–	–	–	–
	6–10 years	−0.294	1.263	−0.008	−0.233	0.816	−2.772	2.184
	11–15 years	−1.047	1.574	−0.025	−0.665	0.506	−4.134	2.040
	16–20 years	1.920	2.071	0.031	0.927	0.354	−2.143	5.983
	>20 years	4.057	2.604	0.068	1.558	0.119	−1.051	9.165
Working hours per week	≤ 40 h per week	–	–	–	–	–	–	–
	41 ~ 48 h per week	1.585	0.659	0.047	2.405	0.016	0.292	2.877
	49 ~ 58 h per week	5.419	1.196	0.088	4.531	< 0.001	3.073	7.765
	≥59 h per week	7.353	1.636	0.085	4.495	< 0.001	4.144	10.562
Number of night shift	0	–	–	–	–	–	–	–
	1–4 times per month	0.983	1.261	0.021	0.780	0.436	−1.490	3.456
	5–8 times per month	1.360	1.123	0.040	1.211	0.226	−0.843	3.563
	>8 times per month	2.975	1.165	0.081	2.553	0.011	0.689	5.261
Work-to-family role conflict	Work-to-family conflict	1.244	0.062	0.51	20.16	< 0.001	1.123	1.365
	Family-to-work conflict	0.319	0.039	0.204	8.223	< 0.001	0.243	0.395

## 5 Discussion

This study, grounded in a cross-sectional design, examined the association between occupational stress and work-family conflict among emergency department (ED) nurses. The mean total occupational stress score was 55.55 ± 16.78 (theoretical range: 22–110), representing 50.5% of the maximum possible score and comparable to previously reported levels among Chinese nurses (23). Both work-to-family conflict and family-to-work conflict were significantly and positively correlated with the total occupational stress score and all its dimensions. Multiple linear regression further identified a bachelor's degree or above, senior professional title, extended weekly working hours, night shift frequency >8 times per month, and work-family conflict as factors independently associated with occupational stress. These findings suggest that occupational stress among ED nurses is closely linked to workload-related factors and is also strongly associated with bidirectional work-family conflict. However, given the cross-sectional design of this study, these associations should not be interpreted as causal relationships.

Role stress theory posits that role conflict arises when individuals face incompatible expectations from multiple roles they occupy simultaneously and provides a framework for understanding the association between work-family conflict and occupational stress. ED nurses are required to continuously invest substantial time, energy, and emotional labor, and are characterized by high workload demands and strong occupational commitment. Such sustained high work demands, when combined with work-family conflict, may be associated with higher levels of occupational stress (23, 24). In this study, both dimensions of work-family conflict emerged as factors independently associated with occupational stress with significant effect sizes, suggesting that work-family conflict is closely related to occupational stress over and above the effects of workload-related factors. This finding indicates that addressing work-family conflict and promoting work-family balance among ED nurses may be an effective approach to mitigating occupational stress, carrying substantial practical implications for the prevention and alleviation of occupational stress (25).

The results of multivariable regression showed that professional title, education level, weekly working hours, and night shift frequency were factors independently associated with occupational stress among ED nurses. These demographic and work-related factors showed significant associations with occupational stress levels.

First, a positive association was observed between weekly working hours and occupational stress. Compared with working < 40 h/week, nurses working 41–48 h, 49–58 h, and ≥59 h/week exhibited increases in occupational stress scores of 1.585, 5.419, and 7.353 points, respectively (*P* < 0.05). Research has demonstrated that excessively long working hours are associated with increased workload (e.g., physical exertion, time commitment, emotional labor), and the high-intensity, high-stress nature of ED work appears to render this association particularly pronounced (23). When work hours persistently exceed the coping threshold of both the individual nurse and their family, higher levels of occupational stress are observed (26, 27).

Second, night shift frequency emerged as another significantly associated factor. In this study, nurses with >8 night shifts per month had significantly higher occupational stress scores than those without night shifts (*B* = 2.975, *P* = 0.011). Night shifts not only disrupt circadian rhythms, impairing sleep quality and recovery capacity, but also further encroach upon the time nurses spend with their families, which may be associated with work-family conflict. Research suggests that night shifts can be regarded as a form of additional workload—their adverse effects are often not fully compensated by organizational support (e.g., night shift allowances, adequate rest periods), and chronic accumulation over time may be associated with persistent occupational stress (28, 29). Notably, night shift frequencies of 1–4 and 5–8 times per month did not show a statistically significant effect, suggesting the existence of a possible cumulative threshold. This finding suggests that night shift frequency >8 times per month may be associated with higher occupational stress. However, given the cross-sectional nature of this study, this association should not be interpreted as a causal or definitive threshold for intervention. Future longitudinal studies are needed to validate this observation before firm scheduling recommendations can be made.

Third, the effects of education level and professional title on occupational stress exhibited a distinctive pattern. Nurses with senior professional titles had significantly higher occupational stress scores compared with those holding junior titles (*B* = 4.744, *P* = 0.012), whereas no significant difference was observed between intermediate and junior titles. This result appears counterintuitive, as senior titles typically confer higher salaries, greater decision-making authority, and increased social recognition (30). However, in the ED environment, senior nurses often assume more complex clinical tasks, teaching responsibilities, managerial pressures, and greater accountability risks, which may increase their workload and responsibility (31). Moreover, senior nurses may experience role overload, frequently shifting among clinical, educational, administrative, and research responsibilities, which may further deplete psychological resources (12). Studies have indicated that senior nurses also hold higher expectations for workplace support and recognition; when actual support falls short of their expectations, the perceived discrepancy may be even more pronounced (32). This finding suggests that interventions for occupational stress should not solely target junior staff—senior nurses equally require psychological support and career development opportunities.

In summary, occupational stress among ED nurses is associated with multiple interacting factors (33–35). Role stress theory provides a useful framework for understanding the associations between long working hours, high night shift frequency, senior professional titles, and occupational stress. Hospital administrators should consider optimizing scheduling practices (e.g., monitoring night shift frequency (>8 shifts per month), regulating weekly working hours) and implementing targeted support and incentive strategies for senior nurses (e.g., flexible management, access to psychological decompression resources). Intervention efforts may need to address both workload reduction and enhanced organizational support to effectively alleviate occupational stress in ED nurses.

It is important to emphasize that due to the cross-sectional design of this study, all associations identified should be interpreted as correlational rather than causal. The regression results indicate factors that are statistically associated with occupational stress, but do not establish that these factors exert causal effects.

### 5.1 Limitations

This study has several limitations that should be acknowledged. First, the cross-sectional design precludes causal inference; the observed associations may be bidirectional. Second, the sample was limited to tertiary hospitals, limiting generalizability to secondary or primary settings. Although the low ICC (0.087) suggested minimal hospital-level clustering, future studies with larger hospital-level samples may consider multilevel modeling to further account for potential contextual influences. Third, self-report measures may introduce social desirability and recall biases, and the ERI questionnaire was used solely as a measurement tool. Fourth, several factors may limit the comprehensiveness and generalizability of the findings: unmeasured confounders (e.g., personality, coping, social support, organizational climate), categorical measurement of night shift frequency without capturing shift pattern complexity, and a sample restricted to emergency department nurses, which may not represent other clinical departments or healthcare professionals. Despite these limitations, this study provides valuable empirical evidence on the associations between work-family conflict and occupational stress among ED nurses in China, with practical implications for nursing management. Future longitudinal studies with objective indicators and organizational-level interventions are warranted.

## 6 Conclusion

This study confirms the significant association between occupational stress and work-family conflict among emergency department nurses. Based on role stress theory, the findings indicate that work-family conflict, as a chronic inter-role stressor, is closely associated with higher occupational stress levels. Notably, work-to-family conflict showed a particularly strong association with occupational stress in this population. The findings suggest that frequent overtime, shift work, and emotional exhaustion are associated with nurses' perception of high workload demands, while simultaneously being associated with reduced family role functioning and emotional wellbeing, which together correspond to higher occupational stress levels. Furthermore, characteristics such as higher education, senior professional titles, long working hours, and high night shift frequency may serve as important markers for identifying populations with higher levels of occupational stress. It is recommended that nursing managers develop targeted intervention strategies—such as reducing unnecessary workload, enhancing workplace recognition and support, and minimizing work-to-family interference—to address occupational stress among emergency department nurses.

It should be noted that, as a cross-sectional study, causality cannot be inferred from these findings. The associations reported here should be confirmed in future longitudinal research.

## Data Availability

The original contributions presented in the study are included in the article/Supplementary material, further inquiries can be directed to the corresponding author.
