# Supplementary material for: The relationship between work-family conflict and occupational stress in emergency department nurses: a cross-sectional correlational study
Source: Front Public Health. 2026 Jul 27;14:1892914. doi: 10.3389/fpubh.2026.1892914 (PMC13454034; doi:10.3389/fpubh.2026.1892914)
Supplement: Supplementary file 1 [file Table_1.docx]

| S1 Collinearity statistics | | | |
| --- | --- | --- | --- |
| Variables | | Tolerance | VIF |
| Age | 20-29 years |  |  |
|  | 30-39 years | 0.218 | 4.591 |
|  | 40-49 years | 0.181 | 5.518 |
|  | ≥50 years | 0.359 | 2.783 |
| Marital status | Unmarried |  |  |
|  | Married | 0.332 | 3.014 |
| Number of children | No |  |  |
|  | Yes | 0.310 | 3.225 |
| Educational level | Associate degree |  |  |
|  | Bachelor's degree or above | 0.909 | 1.100 |
| Professional title | Junior RN |  |  |
|  | Middle RN | 0.586 | 1.705 |
|  | Senior RN | 0.697 | 1.435 |
| Years of nursing experience | ≤5years |  |  |
|  | 6-10 years | 0.263 | 3.806 |
|  | 11-15 years | 0.235 | 4.258 |
|  | 16-20 years | 0.294 | 3.405 |
|  | ＞20 years | 0.168 | 5.945 |
| Working hours per week | ≤40h per week |  |  |
|  | 41~48h per week | 0.841 | 1.190 |
|  | 49~58h per week | 0.868 | 1.152 |
|  | ≥59h per week | 0.911 | 1.098 |
| Number of night shift | 0 |  |  |
|  | 1-4 times per month | 0.438 | 2.284 |
|  | 5-8 times per month | 0.295 | 3.384 |
|  | ≥9 times per month | 0.320 | 3.122 |
| Work-to-family role conflict | Work-to-family conflict | 0.506 | 1.975 |
|  | Family-to-work conflict | 0.525 | 1.906 |

| S2 Significance test of the regression model ^a^ | | | | | | |
| --- | --- | --- | --- | --- | --- | --- |
| Model |  | Sum of squares | Degrees of freedom | Mean square | F | P |
| 1 | Regression | 219875.310 | 20 | 10993.766 | 78.247 | ＜0.001 |
|  | Residual | 213420.508 | 1519 | 140.501 |  |  |
|  | Total | 433295.818 | 1539 |  |  |  |
| a. Dependent variable: Total scores of occupational stresses | | | | | | |

| S3 Test of model fit ^b^ | | | | | |
| --- | --- | --- | --- | --- | --- |
| Model | R | R^2^ | Adjusted R^2^ | Std. error of estimate | Durbin-Watson |
| 1 | 0.712^a^ | 0.507 | 0.501 | 11.853 | 1.945 |
| b. Dependent variable: Total scores of occupational stresses | | | | | |
